# Early Developmental Stress Affects Subsequent Gene Expression Response to an Acute Stress in Atlantic Salmon: An Approach for Creating Robust Fish for Aquaculture?

**DOI:** 10.1534/g3.119.400152

**Published:** 2019-03-18

**Authors:** Nicholas A. Robinson, Hanne Johnsen, Hooman Moghadam, Øivind Andersen, Helge Tveiten

**Affiliations:** * Nofima, Tromsø, NO-9291, Norway; † Sustainable Aquaculture Laboratory - Temperate and Tropical (SALTT), School of BioSciences, The University of Melbourne, Parkville, Victoria, 3010, Australia; ‡ SalmoBreed, Bergen, Norway

**Keywords:** Development, acute stress response, gene expression, DNA methylation, Atlantic salmon

## Abstract

Stress during early life has potential to program and alter the response to stressful events and metabolism in later life. Repeated short exposure of Atlantic salmon to cold water and air during embryonic (E), post-hatch (PH) or both phases of development (EPH) has been shown to alter the methylome and transcriptome and to affect growth performance during later life compared to untreated controls (CO). The aim of this study was to investigate how the transcriptome of these fish responds to subsequent acute stress at the start feeding stage, and to describe methylation differences that might steer these changes. EPH treated fish showed the strongest down-regulation of corticotropin releasing factor 1, up-regulation of glucocorticoid receptor and 3-oxo-5-alpha-steroid 4-dehydrogenase 2 gene expression and a suppressed cortisol response 3 hr after the acute stress, differences that could influence hormesis and be affecting how EPH fish cope and recover from the stress event. Growth hormone 2 and insulin-like growth factor 1 were more strongly down-regulated following acute stress in EPH treated fish relative to E, PH and CO fish. This indicates switching away from growth toward coping with stress following stressful events in EPH fish. Genes implicated in immune function such as major histocompatibility class 1A, T-cell receptor and toll-like receptor also responded to acute stress differently in EPH treated fish, indicating that repeated stresses during early life may affect robustness. Differential DNA methylation was detected in regions mapping <500 bases from genes differentially responding to acute stress suggesting the involvement of epigenetic mechanisms. Stress treatments applied during early development therefore have potential as a husbandry tool for boosting the productivity of aquaculture by affecting how fish respond to stresses at critical stages of production.

Exposure to stressful events during early life stages may result in the development of a variety of metabolic, immune, endocrine and neuro-psychiatric disorders (Kapoor *et al.* 2006; Weinstock 2008; Korosi and Baram 2010; Meaney *et al.* 2007; Love and Williams 2008; Hu *et al.* 2008; Auperin and Geslin 2008; Reul *et al.* 2015). The detailed stress response depends on the nature and effect of the stressor/threatening stimuli, but the initial epinephrine surge, and “fight or flight” response, is generally followed by the activation of the hypothalamic-pituitary-adrenal (HPA) axis, or the functional analog, hypothalamic-pituitary-interrenal (HPI) axis in amphibians and fish (Mateus *et al.* 2017). The release of corticotropin releasing factor (CRF) from the hypothalamic preoptic area induces the synthesis of the pituitary pro-opiomelanocortin (POMC) to be processed into adrenocorticotropic hormone (ACTH). The binding of ACTH to the melanocortin 2 receptor (MC2R) activates the synthesis and release of glucocorticoid (GC) and mineralocorticoid (MC) hormones from the adrenal gland, or the interrenal cells in lower vertebrates (Vander *et al.* 1970). The glucocorticoids and their receptor (GR) play pivotal roles in the response to stressful challenges by regulating a diversity of metabolic, endocrine and immune processes (Korosi and Baram 2010; Seckl 2008; Szyf *et al.* 2007). Stress resilience depends on the proper regulation of basal and stress-induced glucocorticoid levels involving rapid negative feedback control of the HPA/HPI axis (Paragliola *et al.* 2017). Epigenetic mechanisms affecting this feedback in mammals, amphibians and fish seem to involve the regulation of *gr*, *crf1* and *pomc* (Boschen *et al.* 2018; Yehuda *et al.* 2015; Wu *et al.* 2014; Li and Leatherland 2013; Palma-Gudiel *et al.* 2015; Metzger and Schulte 2016; Reul *et al.* 2015).

Cortisol is the main glucocorticoid hormone in fish and exerts its actions through GR and/or the mineralcorticoid receptor (MR) (Baker *et al.* 2013a; Baker *et al.* 2013b; McCormick *et al.* 2008). In addition to its role in the stress response, cortisol is thought to reduce the ability to resist infection or respond to injuries by reducing inflammatory responses and has an important role in energy maintenance through GR signaling (Rose and Herzig 2013; Chatzopoulou *et al.* 2016). Maternally derived cortisol and *gr* transcripts play a key role in developmental programming as demonstrated in zebrafish (Pikulkaew *et al.* 2011; Nesan *et al.* 2012; Nesan and Vijayan 2012; Best *et al.* 2017). The onset of the embryonic synthesis of cortisol varies between fish species, but a cortisol stress-response first develops after hatching in most teleosts (Stouthart *et al.* 1998; Wilson *et al.* 2013).

Despite the lack of an early stress-induced cortisol response, frequent stress treatment during the early development of teleosts has been shown to influence the sensitivity to acute stress at later stages. For example, the plasma cortisol response to acute stress is dampened in 5-month old rainbow trout fingerlings exposed to brief stress at the eye pigmentation, hatching, or yolk resorption stages (Auperin and Geslin 2008). Similarly, unpredictable chronic low intensity stresses during early life stages in sea bass modified the cortisol response to acute stress at the juvenile stage (Tsalafouta *et al.* 2014).

Possession of a functional HPI axis in hatching wild salmon is advantageous in the animals natural habitat at a time when it rises from the gravel and faces many different predators (Hagen *et al.* 2002). In the aquaculture environment there are also many stresses encountered around hatching time (crowding, handling and cleaning) and the response of the fish at this stage could affect survival and performance in aquaculture. Together with the development of the HPI axis, the growth hormone (GH)- insulin-like growth factor 1 (IGF-1) axis seems to be playing a functional role in fish development and differentiation at very early embryonic stages (Besseau *et al.* 2013). Recently we showed that frequent exposure to “mild” stress (cold water and air) during embryonic and/or post-hatch stages affected the methylome and transcriptome of Atlantic salmon and growth performance in later life, providing evidence of hormesis, or an adaptive response to low-level repeated stress (Moghadam *et al.* 2017). Here we extend this study by investigating how Atlantic salmon stressed at early life stages are programmed to respond to acute stresses later in life and whether epigenetic programming during early development is associated with the stress response. The aim of the study was to test how such changes might ultimately affect performance in the aquaculture environment.

## Methods

### Experimental treatments

**Figure 1:**
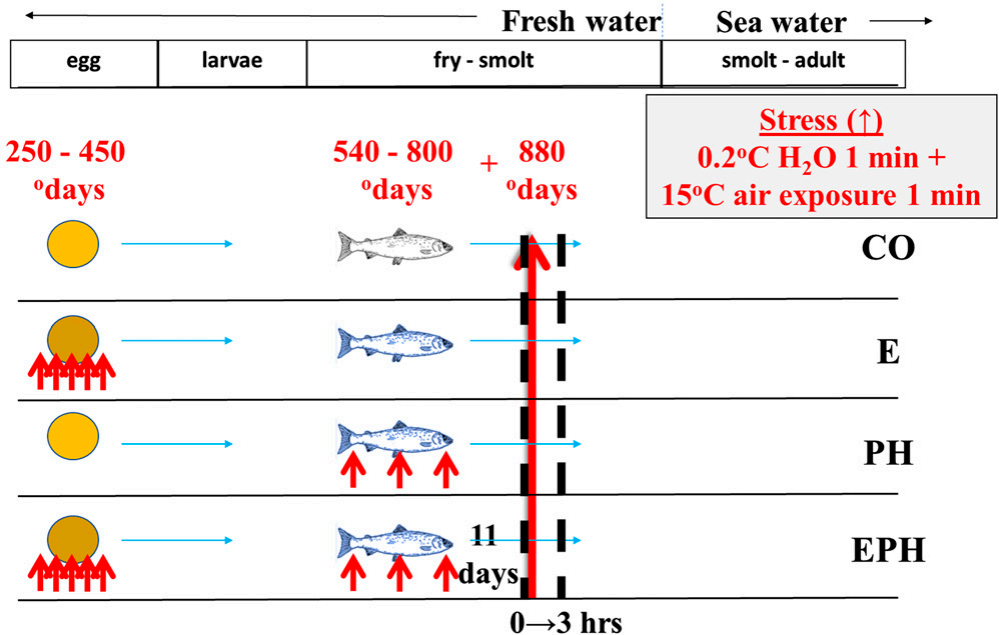
Time course of experimental treatments and sampling for control (CO) embryonic (E) post hatch (PH) and embryonic post hatch (EPH) experimental treatments. Small red arrows indicate application of stresses for the three treatment groups (E, PH and EPH) and the large red arrow indicates the timing and application of the acute stress event to all experimental groups. Dashed black lines show when samples were taken for RRBS and RNA-seq.

Developing Atlantic salmon were treated as described in Moghadam *et al.* (2017). In brief, eggs and milt from a full-sib family were fertilized using standard procedures at the Aquaculture Research Station in Tromsø, Norway. Embryos were divided into four triplicate groups of fish which were *i*: unstressed until 880 ^o^days (*CO*), *ii*: stressed during embryogenesis (*E*), *iii*: stressed during post-hatch (*PH*), and *iv*: stressed during both embryonic and post-hatch stages (*EPH*). The stress treatment consisted of exposure to cold water at 0.2° for 1 min followed by exposure to 15° air for 1 min, 5 times during embryogenesis from 250 – 450 ^o^days and/or 3 times during post-hatch development from 540 – 800 ^o^days (Figure 1). In between the bouts of stress animals were held at 7°. The results from this stress treatment and the resulting body growth after sea transfer are published in (Moghadam *et al.* 2017). The weight of ten randomly picked individuals per replicate was checked prior to start feeding (this was 1 year before the measurement after sea transfer by Moghadam *et al.* 2017).

To test how these different experimental groups responded to an acute stress at a later stage we examined the response of the transcriptome in these fish following a “stress test” at start feeding. To identify associated epigenetic marking that might influence the response of the transcriptome, we looked for differences between the experimental treatments existing in the DNA methylome before the acute stress event. Sixteen thousand fertilized eggs in total were evenly aliquoted to the treatment and control trays. Six fish from each of the *CO*, *E*, *PH* and *EPH* treatment groups were randomly sampled (two fish from each of the three replicate trays per group) to measure baseline gene expression and DNA methylation at 880 ^o^days (designated as 0 hr). This 0 hr baseline gene expression and DNA methylation time point was 11 days (80 ^o^days) after the repeated stress treatments for the PH and EPH groups and 62 days (430 ^o^days) after the repeated stress treatment for the E group. The 0 hr samples were the same as those used to measure gene expression responses and DNA methylation patterns by Moghadam *et al.* (2017). Animals from each group were distributed into special containers housed within a common tank and subjected to acute stress consisting of a 1-minute cold shock and 1-minute exposure to air and sampled at 3 hr post-stress. The post-stress samples were chosen for RNA sequencing to assess the gene expression response to the acute stress (with reference to baseline gene expression in animals sampled before the acute stress).

### mRNA-seq

Samples (whole fish) were stored in RNA later (per manufacturer’s specifications, Ambion). RNA and DNA from 24 individuals from each time point (6 per experimental group) was isolated as described in Moghadam *et al.* (2017). Samples were shipped on dry ice, quality checked using a Bioanalyzer (Agilent) and prepared for sequencing using standard procedures by Zymo Research (San Diego, CA, USA). Total RNA was treated with the Ribo-Zero Magnetic Gold Kit (Human/Mouse/Rat) from Illumina (cat. #MRZG126), and stranded libraries were prepared from rRNA depleted samples using ScriptSeq v2 RNA-Seq Library Preparation Kit from Illumina (Cat. #SSV21106). Sequencing of the 24 libraries of single-end 50 bp reads was performed on an Illumina HiSeq 2500 genome analyzer. mRNA-seq produced on average 64.4 million reads per sample, of which around 60.3 million were mapped to the Atlantic salmon genome (consisting of 19.3 million multiple hits and 41.0 million unique hits on average per sample).

### Reduced representation bisulphite sequencing

Preparation of samples for RRBS sequencing (0 hr) is described in (Moghadam *et al.* 2017). In brief, libraries were prepared from TaqαI and *Msp*I digested gDNA and ligated to adapters containing 5′-methyl-cytosine instead of cytosine according to manufacturer’s instructions (Illumina Inc., San Diego, CA, USA). The ligated fragments were bi-sulfite treated, amplified with PCR and 50 bp of the paired-ends were sequenced on a Illumina HiSeq 2500 genome analyzer.

### Analysis of differential gene expression and DNA methylation

The sequencing reads were trimmed to remove adapters and low-quality base end regions with Trimmomatic (Bolger *et al.* 2014). Trimmomatic was set to: a) remove N’s or low quality sequence (below quality score 15) in leading and trailing bases; b) scan using a 4 base sliding window and trim or cut the sequence when the average quality per base drops below 15 and 3, respectively) exclude any of the trimmed and filtered reads with lengths less than 30 bases. Genomic and transcriptomic short reads were mapped to Atlantic salmon genome assembly GCA000233375.4 ICSASG_v2 (Lien *et al.* 2016) using TopHat version 2.0.8b (Trapnell *et al.* 2009) and Bismark (Krueger and Andrews 2011) with Bowtie1 (Langmead *et al.* 2009) (the latter used to extract methylation status). Default parameters for TopHat were used except for library-type stranded and the *b2* option *sensitive* (which calls a package of pre-set parameters that is passed to Bowtie, Langmead *et al.* 2009). The *scripts*.*count* python program in HTSeq (version 0.8.0, Anders *et al.* 2015) was used to count the reads in the bam file mapping to each feature, ordering the data according to name, skipping all reads with alignment quality less than 10, using *exon* as the feature type, using *gene_id* as feature ID and *union* mode to handle reads overlapping more than one feature. The annotation used to derive the gene counts was created in-house based on RNAseq data from 494 individuals (various life stages and tissues sampled as described in Moghadam *et al.* 2017 and supplied as supplementary data). Differential gene expression was tested using a model for analysis of read coverage data based on the negative binomial distribution (DESeq2 package in R) (Love *et al.* 2014). A principle component analysis of the DESeq2 normalized and regularised-logarithmic transformed read abundance data were conducted to visualize any influence of treatment or time post-acute stress on overall patterns of gene expression.

RRBS methylation data (read coverage and % methylation per base position) was analyzed using logistic regression (methylKit package in R) (Akalin *et al.* 2012). Methylation differences were considered significant where the difference in methylation was >20% and *q*-value <0.1. Areas containing significant DNA methylation differences between treatments were flagged when they occurred <500kb up- or down-stream of differentially expressed genes.

### Cortisol assay and analysis

Cortisol extraction was performed according to de Jesus *et al.* (1991) on 15 samples per treatment (5 per replicate) at 0, 1, 3 and 24 hr post-acute stress. In brief, 0.2 to 0.6 g of embryonic and larval samples were homogenized in 5x (w/v) ice-cold phosphate buffer saline (pH 7.4) with a rotor homogenizer. Cortisol was extracted from 2 × 250 μl of homogenate with 3 ml of diethyl ether. The water phase of the extract was allowed to freeze by placing tubes in -80° and the combined diethyl ether layer was transferred into a new tube. The ether was evaporated by placement of tubes in a 45° water bath for 1 h. Samples were then reconstituted in 250 μl of enzyme immunoassay buffer (EIA buffer Cayman Chemical, MI, USA). Cortisol was quantified using a commercial enzyme immunoassay kit (Cayman Chemical, MI, USA), which was previously used to evaluate zebrafish whole-trunk cortisol measurements (Pavlidis *et al.* 2011). Displacement curves (three different homogenates in serial dilutions 1:10 to 1:75) demonstrated linear parallelism (standard curve *r*^2^ = 0.997 ± 0.001). The recovery of cortisol added to pool homogenates was 94.5 ± 1.2% (mean ± SEM, n = 2).

### Data availability

The GSA Figshare portal was used to upload supplemental files (captions below). All raw sequences were deposited to the NCBI Short Read Archive under the BioProject IDs PRJNA388534 and PRJNA510456. Supplemental material available at Figshare: https://doi.org/10.25387/g3.7586393.

## Results

**Figure 2:**
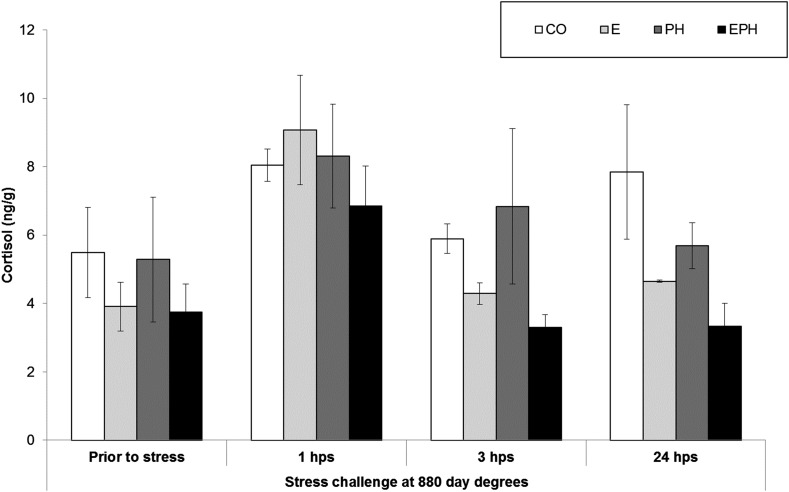
Cortisol concentrations measured in CO, E, PH and EPH treated fish prior to stress and at 1, 3 and 24 hr post-stress (hps).

No significant differences in growth rate or survival were detected between the replicates for any of the treatments used in this study. The four treatment groups showed similar basal levels of cortisol prior to the acute stress at 880 ^0^days and the levels peaked 1 hr after the acute stress in all treatment groups (Figure 2). EPH treated fish tended to have the lowest cortisol levels and returned to pre-stress levels at three hours post-stress. Based on the cortisol levels, preliminary qPCR results (not shown) and the literature, we chose to study the response to acute stress at 3 hr under the assumption that most major differences in gene expression would be occurring two hours after the cortisol peak.

### Genome-wide gene expression changes

**Figure 3:**
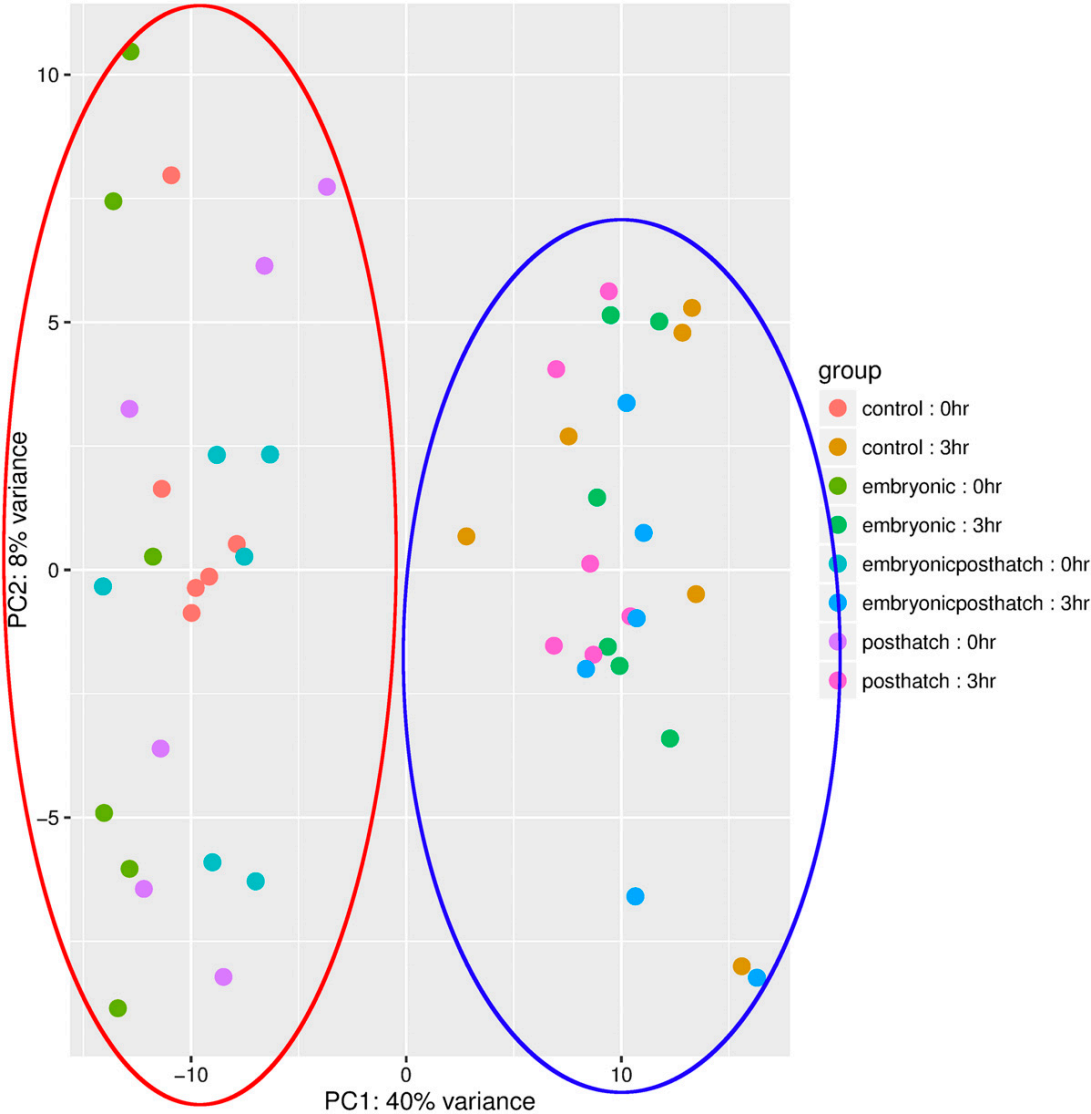
Principle component analysis of treatment *vs.* time after acute stress. The red circle highlights samples taken at 0 hr and the purple circle highlights samples taken at 3 hr post-stress.

To visualize any influence of treatment or time post-acute stress on overall patterns of gene expression, we performed a principle component analysis and showed that samples cluster according to hours post-acute stress (0 hr *vs.* 3 hr, Figure 3). The first component explained most (40%) of the variance. No clustering was apparent for the different treatments.

The expression of large numbers of genes were up- and down-regulated in all experimental groups 3 hr after the acute stress when compared to the pre-stress levels (0 hr) (Supplementary Figure 1). There was a greater trend toward down-regulated gene expression in response to the acute stress in all groups (3746, 2664, 407 and 1517 genes more than onefold down-regulated with *P_adj_* < 0.05 in CO, E, PH and EPH groups respectively) than up-regulated (637, 883, 149 and 854 genes up-regulated respectively with P*_adj_* < 0.05). The largest number of differentially expressed genes were detected for animals previously stressed during embryonic development (E) (Supplementary Figure 1B) while the least were found for animals stressed after hatching (PH) (Supplementary Figure 1C). Early exposure to stress at both embryo and post-hatch stages (EPH) tended to lead to an intermediate acute stress response based on the intermediate number of differentially expressed genes in this group.

As the expression of many genes were affected by the acute stress, we focused in on particular genes that were differentially expressed exclusively in the EPH treated fish compared to the other treatment groups or control fish. Fish treated with an extended period of stress in this way were shown to adaptively respond, and have superior growth, compared to *E* and *PH* stress treatments (Moghadam *et al.* 2017), and therefore we were interested to see how the response to acute stress differs in these fish, particularly for genes affecting the HPI and somatotropic axes, cortisol inactivation and immune response.

### Genes influencing the HPI axis and cortisol inactivation

**Figure 4:**
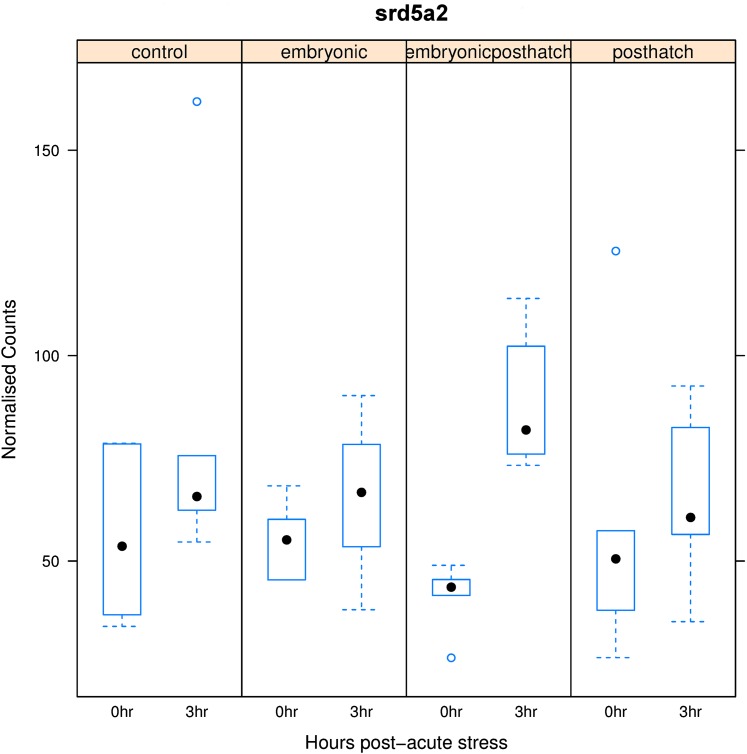
Box plot of changes in the expression of 3-oxo-5-alpha-steroid 4-dehydrogenase 2 (*srd5a2*) for control (CO), embryonic (E), post-hatch (PH) and embryonic post-hatch (EPH) treated fish at 0 and 3 hr post-acute stress.

**Figure 5:**
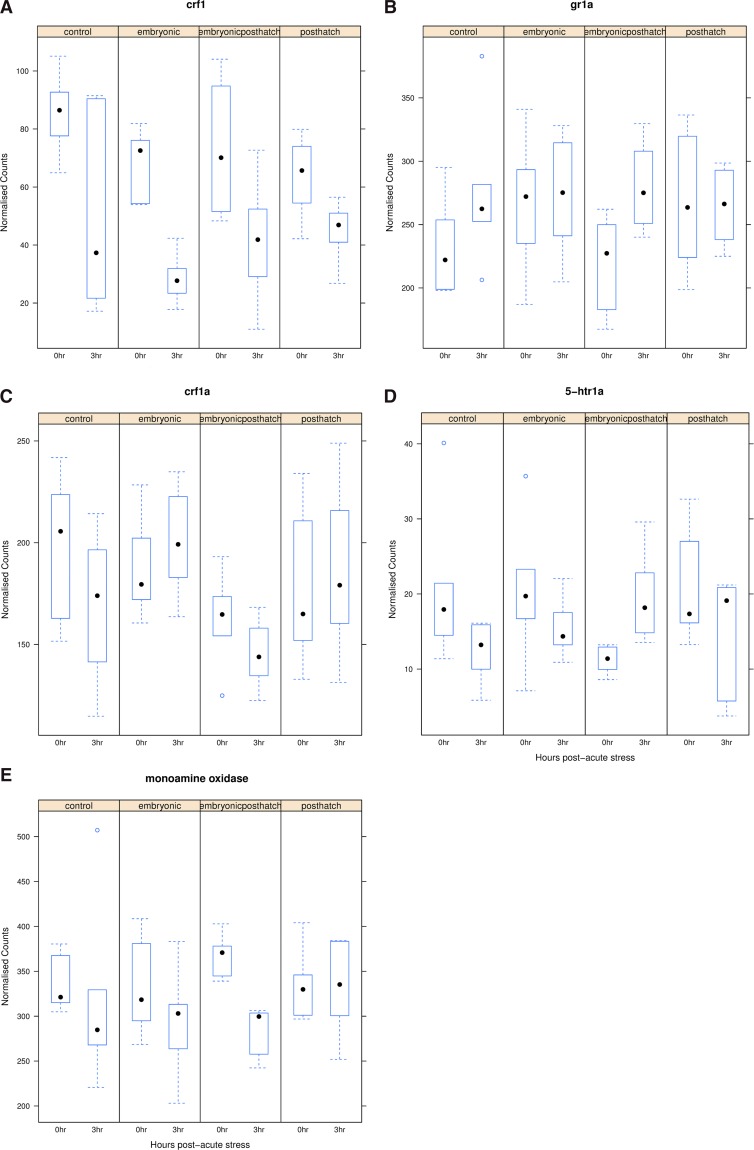
Box plot of changes in the expression of corticotropin releasing factor 1 (*crf1*, A), glucocorticoid receptor 1a (*gr1a*, B), corticotropin releasing factor receptor 1 (*crf1a*, C), 5-hydroxytryptamine receptor 1A (*5-htr1a*, D) and monoamine oxidase (E) for control (CO), embryonic (E), post-hatch (PH) and embryonic post-hatch (EPH) treated fish at 0 and 3 hr post-acute stress.

3-oxo-5-alpha-steroid 4-dehydrogenase 2 (*srd5a2*) was significantly up-regulated in response to acute stress in EPH treated fish, but unresponsive with other treatments (Figure 4). The enzyme encoded by this gene plays a role in metabolism and inactivation of cortisol. The expression of arginine vasopressin-induced 1 (*avpi1*), *crf1* (Figure 5A) and *crf* binding proteins (*crfbp*) and *pomc* A1 and A2 genes was reduced, while the expression of many glucocorticoid and mineralocorticoid receptor genes (*gr1a*, *gr1-like*, *mr1a and mr1blike*, *e.g.*, *gr1a*, Figure 5B) was increased, or stabile, in response to acute stress across all treatments and control. Although insignificant, CO and EPH treated fish tended to show up-regulated *gr1a* expression, while relatively little change in response to acute stress was observed for E and PH treated fish (Figure 5B). Responses to acute stress were insignificant for the *crf* receptors (*e.g.*, *crf1a* Figure 5C) but, *crfr2a* tended to be down-regulated in CO and E, unchanged in PH and up-regulated in EPH treated animals. The brain serotonergic system plays a key role in coordinating stress responses, and the serotonin receptors, or 5-hydroxytryptamine receptors (5-HTR), (*e.g.*, *5-htr1a*, Figure 5D) tended to be down-regulated in expression under all treatments in response to the acute stress, except for *5-htr1b* which was non-significantly up-regulated in the PH and EPH groups (with relatively low levels before the acute stress in EPH treated fish). The enzymatic degradation of serotonin is mainly served by monoamine oxidase, which displayed down-regulated (p_adj_ < 0.01) gene expression in the EPH treatment group (Figure 5E), but did not respond to acute stress in the CO, E and PH treatment groups. In summary, a specific response of the EPH fish to acute stress is suggested by the differential expression of various genes along the HPI axis.

### Response of the somatotropic axis

**Figure 6:**
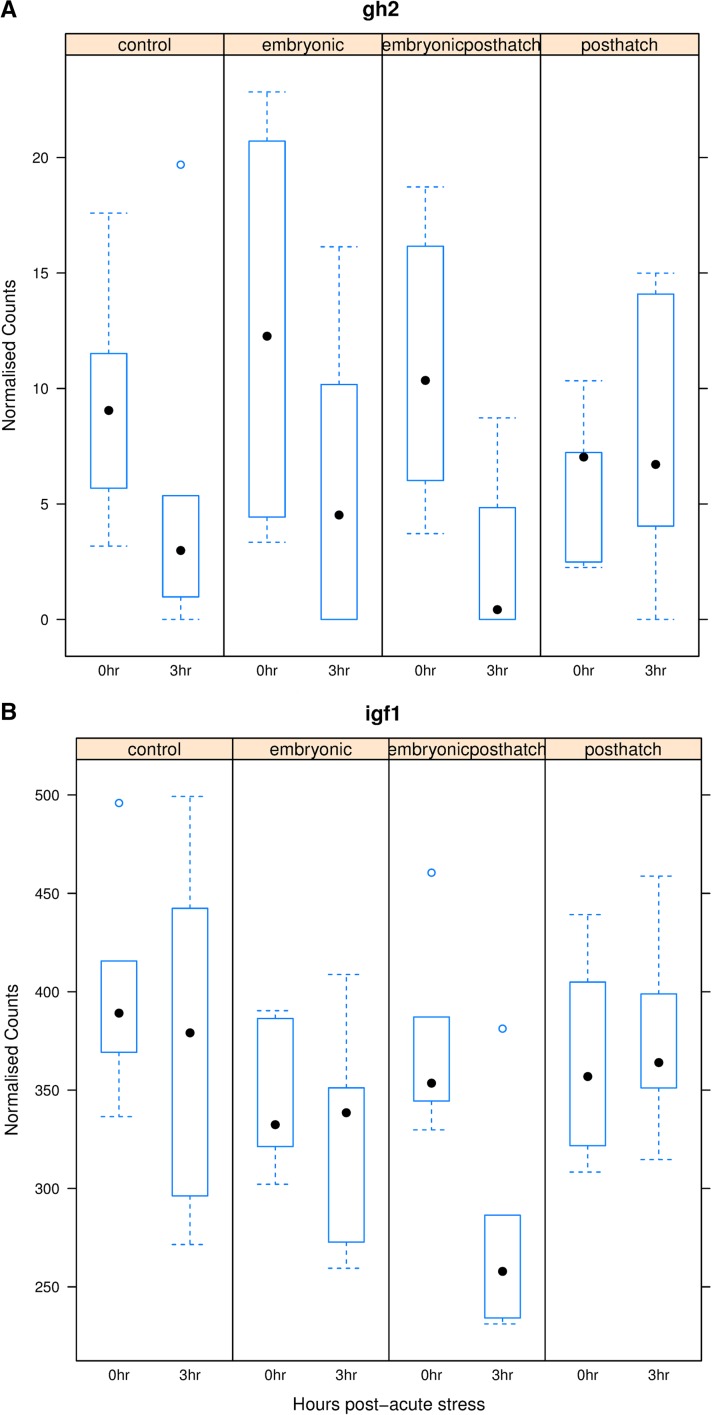
Box plot of changes in the expression of growth hormone 2 (*gh2*, A) and insulin-like growth factor 1 (*igf1*, B) for control (CO), embryonic (E), post-hatch (PH) and embryonic post-hatch (EPH) treated fish at 0 and 3 hr post-acute stress.

Expression of growth hormone 2 (*gh2*) (Figure 6A) and insulin-like growth factor 1 (*igf1*) (Figure 6B) was significantly suppressed for EPH treated fish (*p_adj_* < 0.05), while expression was relatively unresponsive or not significantly down-regulated for the controls and other treatments. Somatostatin 1A (*sst1a*) showed little response to acute stress while the expression of the GH releasing peptide ghrelin tended to be suppressed with acute stress for all treatments. The down-regulation of these genes in the somatotropic axis in response to acute stress after EPH treatment contrasts with the response of genes involved in the HPI axis, wherein *gr* became more strongly up-regulated following acute stress in EPH treated fish.

### Expression differences After repeated stress treatments and response to acute stress

To determine whether genes that were differentially expressed between the E, PH and EPH treated fish (before the acute stress at 0 hr; Moghadam *et al.* 2017) were also differentially expressed in response to the acute stress (3 hr), we performed a Pearson correlation analysis of fold-difference in expression (Supplementary Figure 2). The acute stress had the effect of adding a PH stress treatment such that genes that were up-regulated in EPH compared to E treatment (at 0 hr) were generally the same as those that were up-regulated after acute stress was applied to the E treatment group (0 *vs.* 3 hr, *r* = 0.8, Supplementary Figure 2K). A weaker positive correlation was observed comparing up-regulation in CO relative to EPH treatment with the response to acute stress over 3 hr in CO treated animals (*r* = 0.6, Supplementary Figure 2A).

Genes whose expression was up- or down-regulated in the EPH compared to the PH treatment were often affected in the reverse fashion (down- or up-regulated respectively) when EPH treated animals were subjected to an acute stress (*r*=-0.62, Supplementary Figure 2F). A similar negative correlation was observed for genes that were down- or up-regulated in E treated fish relative to CO compared to the response to acute stress in CO and in E treated animals (*r*=-0.56 and -0.45, Supplementary Figures 2 I and J respectively). These reversals occurred 3 hr after the acute stress is applied and could be a transient response. A possible explanation is that many of these genes are down-regulated due to broad scale switching in favor of genes influencing the acute stress response of the fish. Further research is needed to see whether the expression bounces back or remains suppressed as the animals recover from the acute stress.

### Methylation differences

**Figure 7:**
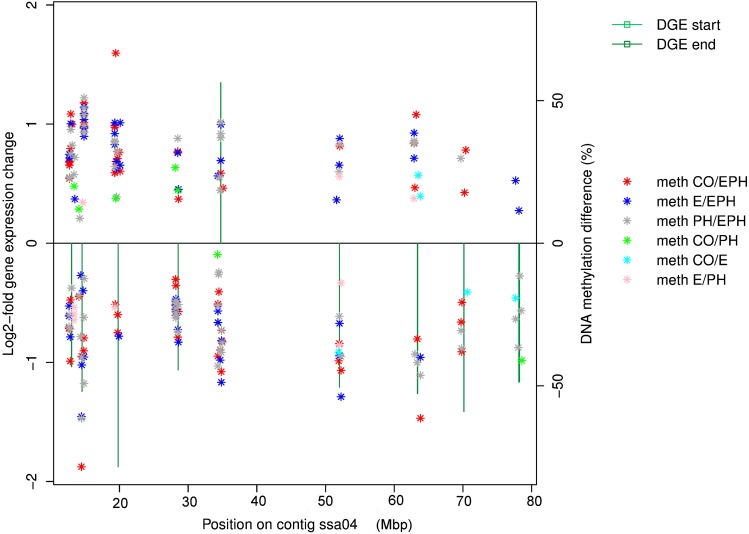
DNA methylation differences between treatments 0 hr pre-acute stress (stars) and gene expression response (from 0 to 3 hr after acute stress, shown as green bars) mapped across chromosome ssa04 for EPH treated fish.

RRBS sequencing yielded on average 42.2 million read pairs, 11.9 million unique CpGs with mapping efficiency of 49%, 6 X coverage and 99% bisulphite conversion rate. Of the 223 genes responding to acute stress in EPH treated animals (log-fold change >1 and *P*_adj_ < 0.01), 156 were found within 500 kb of regions displaying significant differences in methylation associated with treatment. Several interesting DNA methylation and gene expression effects were found to map to ssa04 in particular (Figure 7). Most of these DNA methylation differences (9849 or 55%) involved either increased or reduced methylation with EPH treatment (37%, 34% and 29% compared to PH, E and CO respectively), 45% of the methylation differences occurred in intergenic, 30% in gene, 10% in promoter, 6% in CDS, 6% in five-prime UTR and 3% in three-prime UTR.

**Figure 8:**
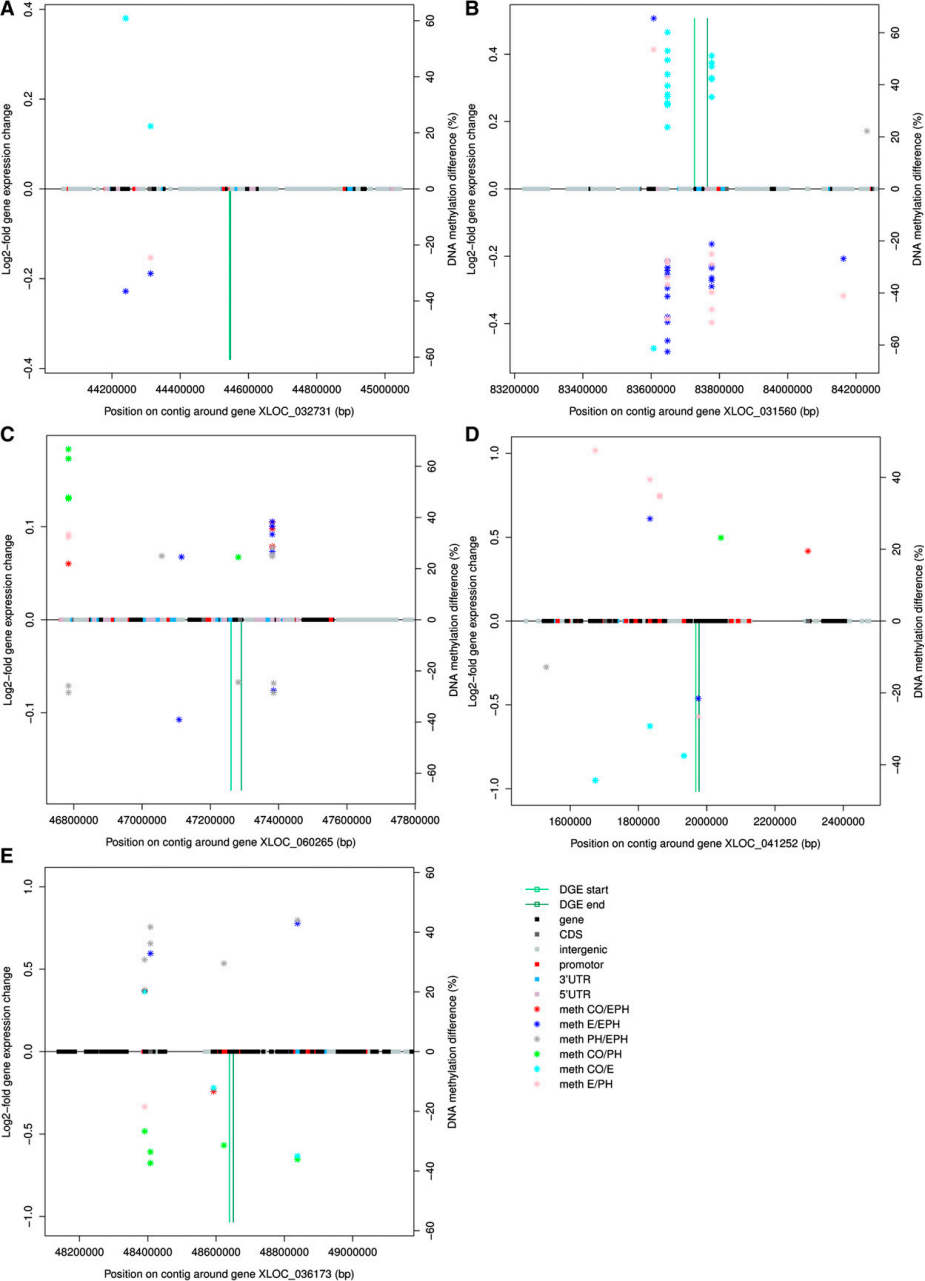
Methylation differences between treatments 0 hr pre-acute stress (stars) and gene expression response (from 0 to 3 hr after acute stress, shown as green bars) for mapped to positions in the genome for EPH treated fish. Significant gene expression changes with acute stress and DNA methylation differences with treatment are shown for *pomc-a1* (XLOC_032731) (A), *gr2* (XLOC_031560) (B), serotonin transporter solute carrier family 6 member 4 (XLOC_060265) (C), *gh2* (XLOC_041252) (D) and around the *hsp70* gene (XLOC_036173) (E).

Of the differentially expressed genes in the HPI axis, the down-regulated expression of *pomc1a* in the CO, E and EPH fish, but up-regulation in PH fish, might be influenced by DNA methylation in the dyactin subunit (*dctn1*) and Wolf-Hirschhorn syndrome candidate (*whsc1*) located down-stream on ssa09. The methylation levels were significantly reduced with EPH or PH treatments relative to E treatment, but significantly enhanced with E treatment relative to controls (Figure 8A). *gr2* is positioned ∼400 kb from *pomc-a1* and showed up-regulated expression in all groups. This might be associated with the DNA methylation in up- and down-stream regions of the promoter and intergenic region of the heat shock 70 protein 4 (*hsp70A4*) gene, which is located 22 kb from *gr2* on ssa09. DNA methylation was significantly reduced with EPH or PH treatments relevant to E treatment and methylation was significantly enhanced with E treatment relative to controls (Figure 8B). It therefore seems unlikely that the enhanced DNA methylation for the E treated fish is connected to the differential gene expression detected for *gr2*. Other factors are possibly influencing the regulation of *gr2* expression. DNA methylation was found to be significantly higher in a region upstream of the serotonin transporter solute carrier family 6 member 4 (*slc6a4*) on ssa20 in EPH treated fish compared to CO, E and PH treated fish (Figure 8C), while the methylation of a down-stream region was significantly increased by PH treatment compared to E, C and EPH.

Of the differentially expressed genes in the somatotrophic axis, significant differential DNA-methylation with treatment (*q*-value < 0.1) was also found in various gene and promoter regions up- and down-stream of *gh2* on ssa12 (Figures 6A and 8D). These differentially methylated genic regions code for zinc finger proteins (*zf*879-like, *zf* 34-like, *zf* 558-like, *zf* 501-like and *zf* 239-like) and one uncharacterized transcript. A tendency for higher methylation for PH treated than E treated animals (pink stars) and less methylation for E compared to CO treated animals (light blue stars) was detected at sites upstream of the gene (Figure 8D). A single base position in the *gh2* gene showed higher methylation in E treated animals (E/EPH blue and E/PH pink stars, Figure 8D). A similar strong pattern of methylation change between treatments was detected for *gr2*, but this time with the E treatment having significantly reduced methylation relative to CO, and the EPH treatment having significantly higher methylation in this position than E and PH treatments. However, points with methylation differences between treatments were scattered around *gh2* and *gr2*, with no distinct CpG islands of methylation (*e.g.*, *gh*2, Figure 8D).

The *hsp70* on ssa10 is another gene whose expression was significantly down-regulated in response to acute stress in EPH treated fish, but was relatively unresponsive for fish of other treatment groups. Two areas surrounding the gene showed distinct patterns of changed methylation with treatment (*q*-value < 0.1, Figure 8E). One of these areas was located in a 3′ UTR for transferrin-like domain ∼200 kb up-stream and one in a 3′ UTR for lamin nuclear inner membrane filament B2 ∼200 kb down-stream. Methylation in the upstream 3′ UTR and gene was reduced and increased with PH and EPH treatment respectively.

**Figure 9:**
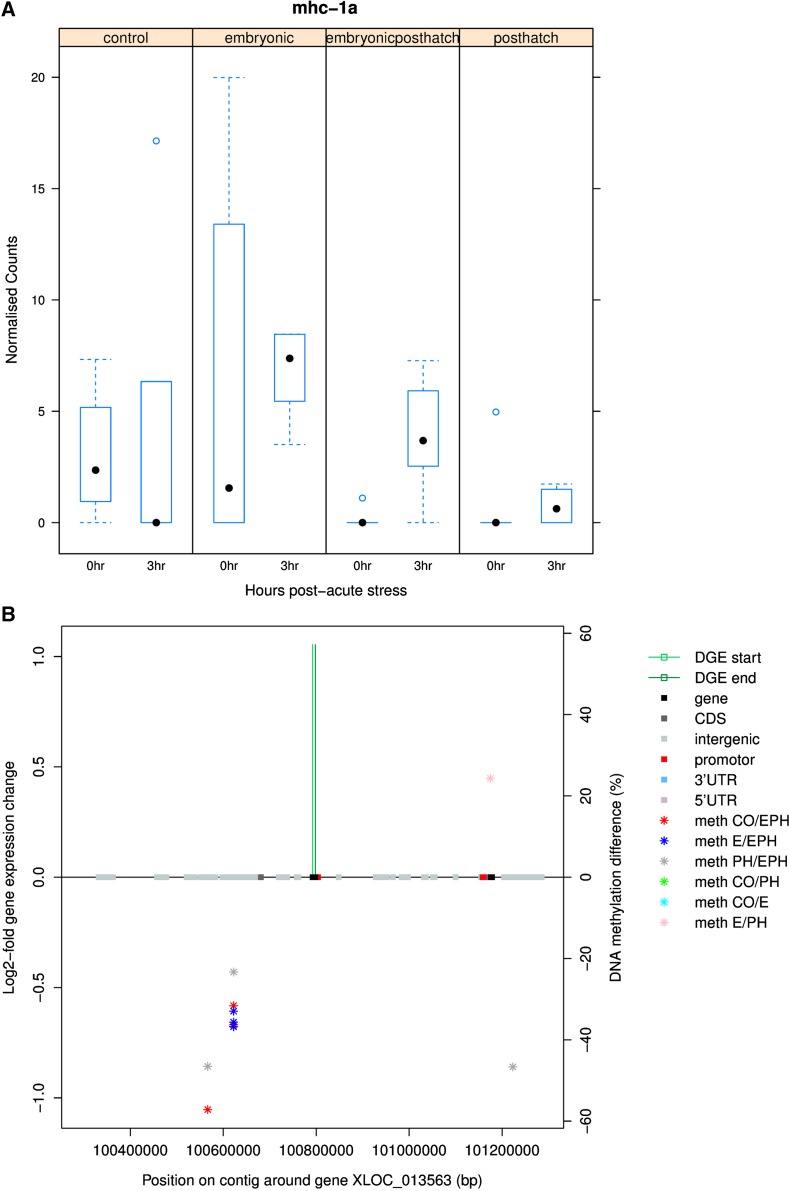
Box plots of changes in the expression of MHC class 1A (transcript XLOC_013563) for control (CO), embryonic (E), post-hatch (PH) and embryonic post-hatch (EPH) treated fish (A) and map of methylation differences between treatments 0 hr pre-acute stress (stars) (B). The gene expression response for EPH treated fish (from 0 to 3 hr after acute stress) is shown as green bars positioned at the start and end of the gene transcript on the methylation map.

DNA methylation in proximity to several differentially expressed immune genes was also noteworthy. MHC class IA core region is up-regulated with acute stress in EPH treated animals (*p_adj_* < 0.05) and intergenic regions ∼200kb upstream show higher methylation with EPH treatment (Figure 9). T-Cell Receptor gamma (*tcr*γ) was significantly down-regulated after acute stress in EPH treated animals (*p_adj_* < 0.01, unchanged for CO, E and PH) and an uncharacterized gene region ∼100kb downstream was found to contain higher DNA methylation with PH and EPH treatment (Supplementary Figure 3). Toll-like receptor 3 (*tlr3*) was more than onefold down-regulated with acute stress in EPH treated animals (*p_adj_* < 0.01) and various genes up- and down-stream showed patterns of increased or decreased methylation depending on treatment comparison (Supplementary Figure 4). Immunoglobulin H (*igh*) locus B (one transcript) and locus A (two transcripts) were more than onefold down-regulated with acute stress in EPH (*p_adj_* < 0.01) and E treated animals (*p_adj_* < 0.05). Differences in methylation in proximity to the *igh* locus B gene mapped to intergenic, promoter, CDS and gene regions (Supplementary Figure 5). The ATP-binding cassette (*abc*) transporters were up-regulated with acute stress in EPH treated animals (*p_adj_* < 0.01). Intergenic regions showing higher methylation with PH treatment and lower levels of methylation with EPH treatment were identified up- and down-stream respectively from the gene (Supplementary Figure 6).

## Discussion

### Response of the HPI axis to acute stress

The present study documented a relatively weak cortisol response to acute stress in Atlantic salmon at the start feeding stage compared to the ten times greater response that was recently shown in the fully formed adult (Höglund *et al.* 2017). The tendency for lower cortisol levels throughout the experiment in EPH treated fish, and the reduction of cortisol to below pre-stress levels by 3 hr post-acute stress in EPH treated fish, is an indication that the EPH treatment has modified the overall coping style of the fish relative to other treatments. The suppression of cortisol expression in EPH treated fish, and the long-term effects of this modified response as the fish encounters other stresses in the culture environment, might also be linked to the enhanced growth rate of EPH treated fish detected by our associated study when the fish were transferred to sea (Moghadam *et al.* 2017).

One possible way that EPH treatment might be modifying the coping style of the fish and suppressing the cortisol response to acute stress is by promoting the expression of genes that inactivate or metabolize cortisol. One such gene is *srd5a2*, of which two isoforms were detected in salmon, one of which is significantly up-regulated in response to acute stress in EPH treated fish but not in fish receiving other treatments (Figure 4). *srd5a2* plays a role in the metabolism and inactivation of cortisol via 5α-reduction of the cortisol A ring double bond and is also responsible for 5α-reduction of testosterone (Fisher *et al.* 1978). Inhibition of the gene has been shown to augment cortisol action (Nasiri *et al.* 2015).

Down-regulation of *avpi1*, *crf* 1, *crf* binding protein 1a and 1b transcripts in response to acute stress in all treatment and control groups suggests that the brain hypothalamus-pituitary connection was functioning at the start feeding stage of development and that a negative feedback loop was activated by cortisol at 3 hr post-stress. This was further evidenced by the down-regulated expression of both *pomc* a1 and a2 in response to acute stress in E and EPH treated and control fish. *pomc-b* was previously found to respond in the same way in juvenile salmon at the parr stage one hour after acute stress (Madaro *et al.* 2015).

### Modulation of the growth and stress axes in response to repeated stress During early development

In terms of the overall number of genes responding to later life acute stress, PH treated fish responded the least, E the most and EPH gave an intermediate response. This suggests that the duration and timing of the stress treatments effectively modulates the epigenetic programming of the fish and primes the subsequent acute stress response. Cortisol binds and acts on both the GR and MR, which play important roles in the feedback and regulation of cortisol secretion. The *gr* expression was shown to be a consistent indicator of stress axis activity in sea bass (Terova *et al.* 2005). Many of the genes that are exclusively responding in EPH treated individuals were found to have putative functions that could contribute to an adaptive response in later life. These subtle differences in the response to acute stress between treatments, as detected for *crfr1* and *gr*, could be affecting how the fish copes and recovers from the stress event, and influencing the hormesis with the EPH treatment that was observed in our associated study (Moghadam *et al.* 2017). The *gr* gene tended to be up-regulated in response to stress in CO and EPH treated fish but unchanged in E and PH treated fish (Figure 5B). Life-long stressors such as repeated handling stress are known to lead to larger numbers of GR receptors in the rodent brain and increased methylation of the promoter region of this gene (Anacker *et al.* 2014). We found that DNA methylation in areas mapping around these genes was associated with the timing and duration of the chronic stress experienced during early development (Figure 8 A and B). Vindas *et al.* (2016) also showed that early life exposure to chronic stress reduces the response to acute stress and proposed that this could be inducing habituation to the aquaculture environment.

*Gh*1 transgenic Atlantic salmon not only demonstrated substantially faster growth rates than non-transgenic animals (Du *et al.* 1992), but also displayed elevated metabolic rate and improved oxygen delivery (Don Stevens and Sutterlin 1999; Cook *et al.* 2000). The salmonid *gh*1 and *gh*2 genes have distinct regulatory modes and are thought to function differently or at different times of development (von Schalburg *et al.* 2008). The suppression of *gh*2 and *igf*1 expression in response to acute stress in EPH treated animals might be contributing to the enhanced growth rate of the EPH treated fish detected in our associated study (Moghadam *et al.* 2017). The up- and down-stream DNA methylation differences that were detected in EPH fish suggest that epigenetic effects could be steering this differential response. The precise influence of reduced or raised DNA methylation in areas around these genes should be cautiously interpreted. The levels of methylation were measured at 0 hr immediately before animals were subjected to the acute stress. Differences in the extent of DNA methylation in these areas could possibly affect the baseline level of gene expression before the acute stress, the extent of the gene expression response to the acute stress, the direction of expression change in response to the acute stress or might not have any effect at all on the response to acute stress. We restricted our analysis to <500kb up- or down-stream of differentially expressed genes, but it could be that there are other CpG regions outside of these windows where levels of DNA methylation are associated with differential gene expression (either causative or type I error associations). Further experimentation, for example involving DNA methylation editing (Liu *et al.* 2016), would be needed to test whether DNA methylation at these <500bp up- or down-stream positions are actually influencing the response of these genes to acute stress. The contrasting responses of the stress and growth response axes in EPH treated fish is noteworthy: *gh*2 and *igf*1 are more strongly down-regulated at 3hrs post-stress (Figures 6 A and B), whereas, expression of the *gr* gene is more strongly up-regulated following acute stress in EPH treated fish than fish of other treatments (Figure 5B). This result could indicate a stronger switching away from growth and toward coping with stress following stressful events in EPH treated fish, a strategy that might benefit the recovery of the fish and ultimately provide a better long-term growth response, but more data on temporal changes in the expression of genes in the somatotropic and HPI axes in EPH treated fish is needed to test this hypothesis. A similar pattern has been observed before in response to chronic stress in salmon and sea bass (Moghadam *et al.* 2017; Fokos *et al.* 2017; Vindas *et al.* 2016). Up-stream positive and negative feedback regulators of *gh* expression, such as gonadotrophin-releasing hormone and preproghrelin-1 and 2 (Lim *et al.* 2014), tended to show reduced expression in all treatment and control groups. Three transcripts annotated as *igf*-1 were down-regulated in EPH treated animals and up-regulated in PH treated animals (n.s., like the response of *gh*2). *igf*-1 can act as a negative or positive feedback regulator of *gh* expression (Romero *et al.* 2012) and is an important mediator of the metabolic response to acute stress (Wiseman *et al.* 2007).

Serotonin is believed to play an important role in coordinating the stress response of fish and other animals (Backstrom and Winberg 2017) and has been found to be involved in switching the allocation of energy during acute stress from functions affecting growth and reproduction to functions affecting the animal’s ability to cope with stress (Andrews *et al.* 2015), inhibiting the release of growth hormone (Yu *et al.* 2008) and regulating the production of ATP from glucose by stimulating glycogen breakdown (Conde-Sieira *et al.* 2010). Glucosensing systems are deregulated under stress and unable to respond to changes in glucose levels (Conde-Sieira *et al.* 2010). The expression of the serotonin receptors was generally down-regulated under all treatments in response to the acute stress, but EPH treated fish showed suppressed *5-htr1a* expression relative to the other groups before the acute stress was encountered. In comparison, Vindas *et al.* (2016) reported increased *5-htr* transcript levels in the brain stem and hypothalamus of salmon one hour after acute stress, and the catabolism of serotonin was significantly higher in control fish than in fish previously exposed to unpredictable chronic stress. Consistently, the serotonin degrading enzyme monoamine oxidase did not respond to acute stress in the CO, E and PH treatment groups, but was down-regulated in expression in the EPH treatment groups. Previous studies showing that chronic stress treatment during early life influences growth rate as fish enter the sea (Moghadam *et al.* 2017; Vindas *et al.* 2016) could be partly explained by some form of mitigated monoamine response.

### Repeated stress During early development programs the acute stress response of heat shock protein 70

Heat shock protein (*hsp*) expression is up-regulated when organisms come under stress and the HSP’s are known to play critical roles in the folding and assembly of proteins, functioning of the immune system, apoptosis and inflammation, all of which help reduce trauma and physical stress (Roberts *et al.* 2010). This defense mechanism is activated in the earliest developmental stages of fish (*e.g.*, Wu *et al.* 2018). Expression of *hsp70* was more than onefold down-regulated 3 hr after acute shock in EPH treated fish (but not in fish of the other treatments) and 3′UTR regions 200 kb up- and down-stream showed increased DNA methylation with EPH treatment. The response of *hsp*70 expression to stress differs depending on the timing, duration, type of stress and species (Takle *et al.* 2005; *e.g.*, Moniruzzaman *et al.* 2017). Repeated embryonic thermal stress has also been found to reduce or down-regulate subsequent *hsp*70 responses to acute heat shock in whitefish (Whitehouse *et al.* 2017). Short-term heat exposure of the early Atlantic cod blastula, and long-term heat exposure to the embryo, both result in up-regulation of *hsp*70 (Skjærven *et al.* 2011). *hsp*70 is upregulated during and after metamorphosis in Senegalese sole and embryos incubated at lower temperatures contained higher levels of *hsp*70 (Campos *et al.* 2013). Both cold- and heat-shock induces expression of *hsp*90 in zebrafish larvae, while *hsp*70 and *hsp*70-like expression gradually increases in cold-exposed zebrafish larvae (Long *et al.* 2012). *hsp*70 is transiently elevated in trout plasma after heat shock (Faught *et al.* 2017). The modulated response of *hsp70* gene expression to acute stress is another indication that the stress coping style has been modified by the EPH treatment.

### Changes in immune system genes

Hormesis elicited by repeated stress, as possibly experienced by EPH treated fish (Moghadam *et al.* 2017), could in some instances suppress the response of the immune system and make the animal more vulnerable to disease (Tort 2011). Equally, such exposure to mild repeated stress could pre-prepare or enhance the response of the immune system and allow the animals to cope and perform better in the face of infectious disease. Decreased *gr* responsiveness with stress-related disorders reduces the ability of mammals to resist disease (Raison and Miller 2003). We observed that the response of *gr* to acute stress was dampened by the E and PH treatments, but that the EPH treated fish responded in a similar fashion to the CO fish. This difference in the responsiveness of the stress axis could therefore be influencing the immune response of the fish.

Various immune responsive genes were differentially expressed in response to acute stress in EPH treated fish. Significant differential DNA methylation associated with treatment was also detected up- and/or down-stream of these loci. Specific alleles of *mhc* I and II have been found to be associated with resistance to infectious salmon anemia in Atlantic salmon (Kjøglum *et al.* 2006; Grimholt *et al.* 2003) and salmon resistant to ISAV infection show differential expression of *mhc* IIB in the spleen (Dettleff *et al.* 2017). The *tcr* recognizes antigens bound to *mhc* (Guan *et al.* 2016). Other pentaxin type proteins like *tcr* (*e.g.*, C-reactive protein, which responds to interleukin-6 secretion by the T-cells and binds to dead or dying cells to activate the complement system; Scheid *et al.* 1994) were also found to be down-regulated in response to acute stress in EPH treated fish but unchanged in CO, E and PH fish. The *tlr3* gene plays an important role in the recognition of viral particles (*e.g.*, salmonid alphavirus subtype 3), and along with the RIG-I-like receptors, uses interferon regulatory factors (3 and 7) to produce interferon-a2 (Xu *et al.* 2016). Methylation of *abc* and differential expression of *tlr* signaling pathway genes have been detected in cold-shocked zebrafish larvae and embryos (Long *et al.* 2012; Han *et al.* 2016). Programming of immune response genes could have important implications for the robustness of the fish when exposed to disease in aquaculture, and the effect of the EPH treatment on the gene expression response and effect of these genes therefore warrants further investigation.

### Conclusions and relevance for aquaculture

A functional HPI axis was found to exist in Atlantic salmon at 880-^o^days after fertilization. EPH treatment was found to influence the responses of the HPI and somatotropic neuroendocrine axes to acute stress. Our results suggest that EPH fish could be better able to switch between stress response and growth, and that immune function may also be differentially affected in EPH treated fish. Levels of DNA methylation measured immediately before the acute stress were found to be associated at closely mapping positions to genes that were up- or down-regulated in expression in response to the acute stress in some instances.

The results highlight that the history of the fish is important. It is often assumed that fish return to normal resting condition 24 hr after a stress, whereas our evidence suggests that the fish may never fully recover. It is probably not a good strategy to expend energy on growth in times of stress and the strategy adopted by EPH treated fish when acutely stressed might lead to better recovery and growth in the long-term. However, it must be remembered that these are dynamic responses and the situation could change over time.

The unique immune gene response to acute stress detected in EPH treated fish suggests that the EPH treatment could also have implications for robustness and warrants further investigation. Treatments that are able to modify stress sensitivity therefore could have potential for boosting survivability and productivity at critical stages of production, such as when Atlantic salmon are transferred to cages in the sea when they are particularly stressed and vulnerable to disease.
